# Primary Duodenal Periampullary Adenocarcinoma: An Uncommon Presentation

**DOI:** 10.7759/cureus.14323

**Published:** 2021-04-06

**Authors:** Rasiq Zackria, Mahesh Botejue, Andrew W Hwang

**Affiliations:** 1 Internal Medicine, University of California, Riverside School of Medicine, Riverside, USA; 2 Graduate Medical Education, Riverside Community Hospital, Riverside, USA

**Keywords:** periampullary carcinoma, duodenal neoplasm

## Abstract

Periampullary carcinoma is a broad term used to define the group of carcinomas arising from the head of the pancreas, the distal common bile duct, and the duodenum. It is clinically important to differentiate ampullary from periampullary carcinoma as this can affect resectability and prognosis. Atypical left-sided chest pain is an atypical presentation of periampullary duodenal adenocarcinoma. A 58-year-old man presented with a two-month duration of worsening intermittent, atypical, migratory left-sided chest pain. Imaging studies were unremarkable; however, endoscopic evaluation demonstrated a duodenal mass. While most periampullary carcinomas are generally curable with pancreaticoduodenectomy, if left untreated, these tumors are uniformly fatal.

## Introduction

Periampullary carcinoma is a broad term used to define the group of carcinomas arising from the head of the pancreas, the distal common bile duct, and the duodenum. Overall, periampullary carcinoma accounts for more than 30,000 deaths per annum in the United States. This neoplasia should be differentiated from ampullary carcinoma, as periampullary carcinoma tumor is anatomically centered in the region of the ampulla of Vater and the duodenal portion of the bile and pancreatic duct. It is clinically important to differentiate ampullary from periampullary carcinoma as this can affect resectability and prognosis. While most periampullary carcinomas are generally curable with pancreaticoduodenectomy, if left untreated, these tumors are uniformly fatal.

## Case presentation

A 58-year-old man with no reported medical history presented for worsening intermittent, atypical, migratory left-sided chest pain, and jaundice of two-month duration. He reported recent development of upper respiratory infection symptoms without full recovery. He reported associated dyspnea but denied other symptoms of fever, chills, palpitations, nausea, emesis, decreased appetite, unintentional weight loss, abdominal pain, and change in bowel habits. Also, he denied any prior similar episodes and reported a negative cardiac workup for ischemia one month ago. At that time, ultrasonography (US) of the gallbladder and computed tomography (CT) of the abdomen and pelvis without contrast were unremarkable. 

**Figure 1:**
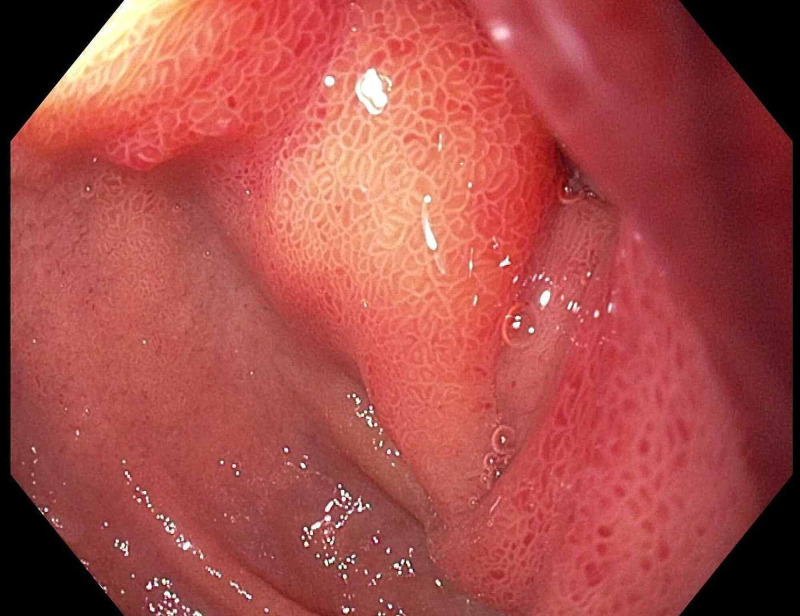
Esophagogastroduodenoscopy demonstrating the duodenal mass in the periampullary region.

**Figure 2:**
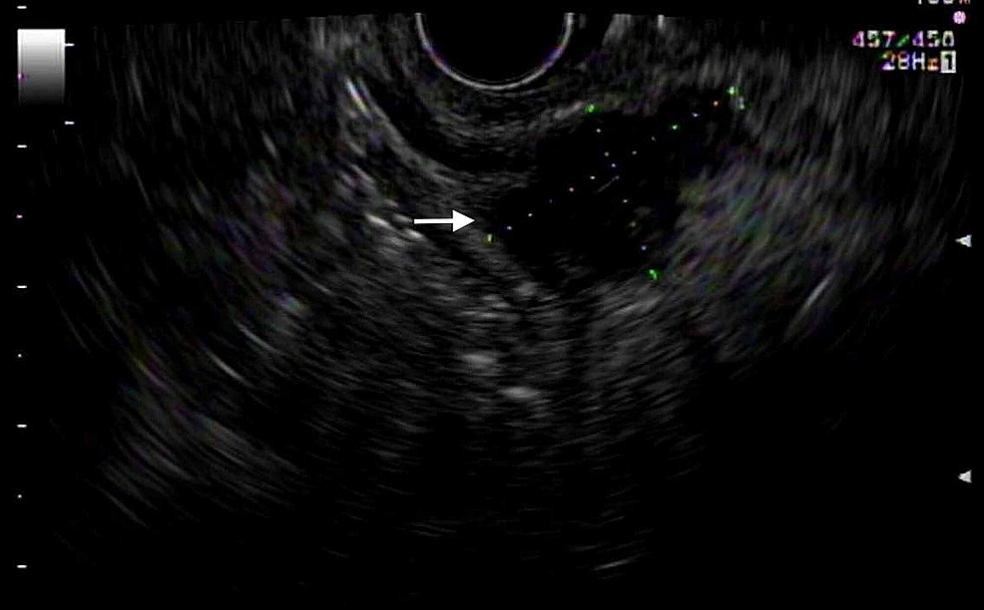
Endoscopic ultrasound showed a hypoechoic circumferential mass measuring 25 mm by 15 mm in maximal cross-sectional diameter; there was sonographic evidence suggesting invasion into the muscularis propria, as indicated by the white arrow.

Physical examination was unremarkable except for jaundice. Imaging studies including US of the abdomen, CT of the abdomen with and without contrast, and magnetic resonance cholangiopancreatography (MRCP) did not demonstrate any abnormalities. Esophagogastroduodenoscopy (EGD) and endoscopic ultrasound (EUS), pursued due to persistent jaundice, revealed a duodenal mass; subsequent biopsy demonstrated poorly differentiated invasive adenocarcinoma (Figures 1-2).

**Figure 3:**
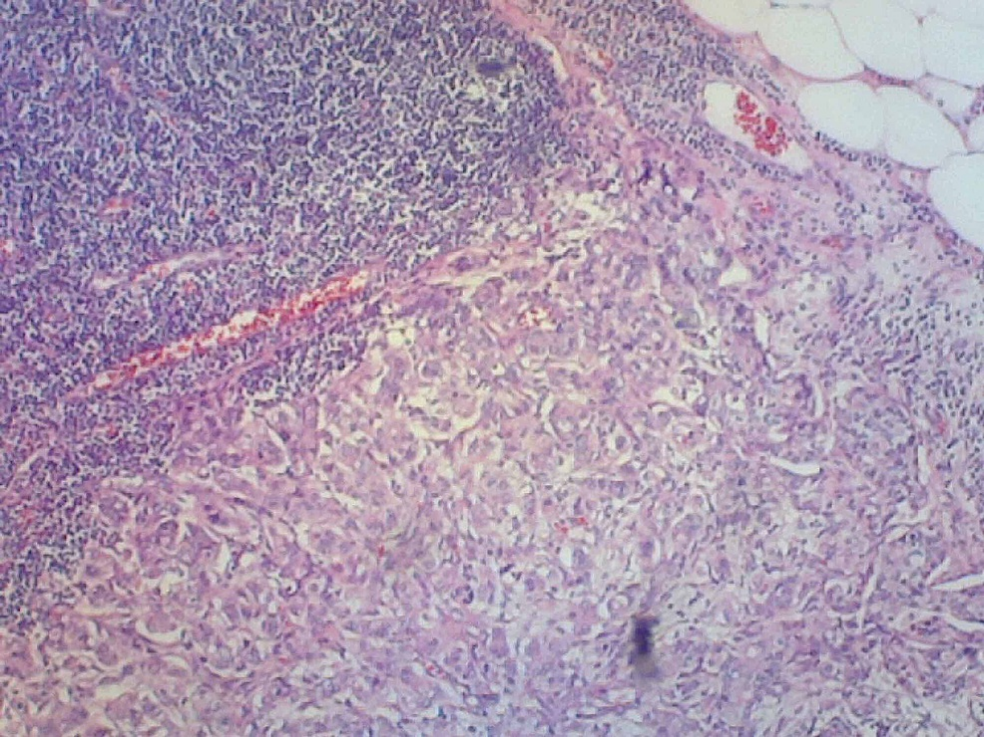
Hematoxylin and eosin (H&E) stain pathology demonstrating normal lymph node in the upper left with metastatic spread in the lower portion of the image.

**Figure 4:**
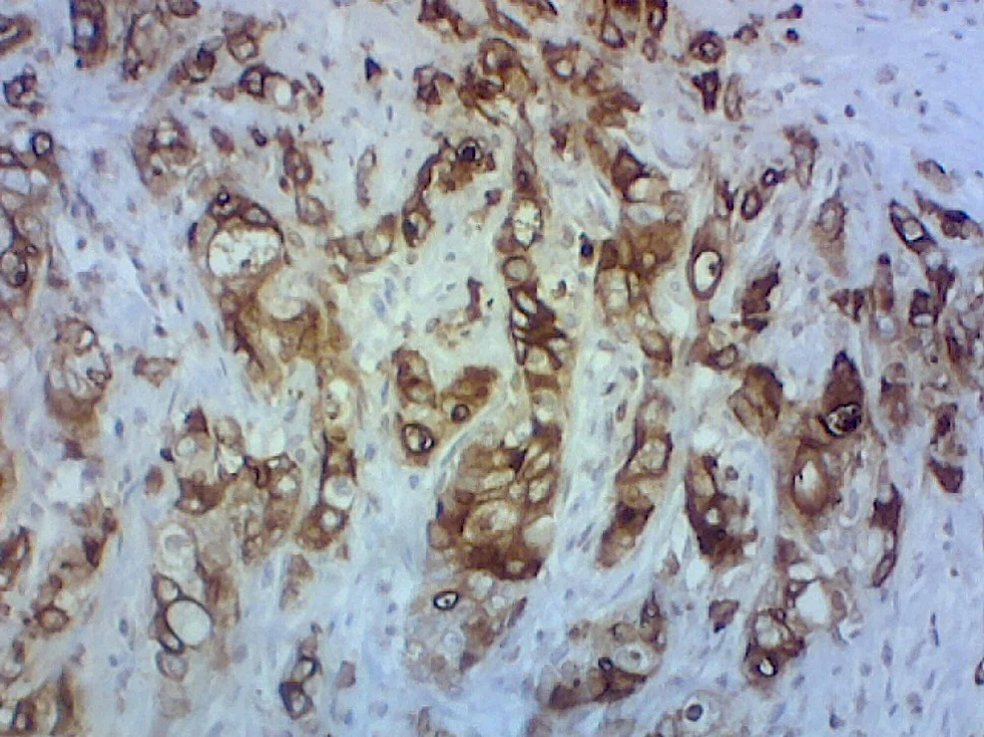
Mucin 5AC (MUC-5AC) immunohistochemical stain that is positive, consistent with the diagnosis of periampullary duodenal adenocarcinoma.

He underwent an uncomplicated pancreaticoduodenectomy and lymph node resection. Gross specimen evaluation confirmed the diagnosis of periampullary duodenal adenocarcinoma with extension through the serosal surface of the duodenal wall with lymphocytic spread (as seen in Figure 3) and immunohistochemical markers showing positive staining in the tumor cells with cytokeratin (CK)-7, CK-17, mucin 1 (MUC-1), and mucin 5AC (MUC-5AC) (Figure 4). He was discharged home with outpatient oncology follow-up for further management. 

## Discussion

Duodenal adenocarcinoma is a rare cancer, representing only 0.35% of all tumors of the digestive tract. While the duodenum accounts for less than 10% of the total length of the small intestine, it makes up 25%-45% of all small bowel malignancy [1]. Periampullary tumors include pancreatic adenocarcinoma, distal bile duct, and periampullary duodenal carcinomas and are described as arising within 2 cm of the major duodenal papilla [1,2]. In contrast, ampullary carcinomas are primarily in the region of the ampulla of Vater accounting for about 0.2% of tumors of the gastrointestinal tract [1]. Clinically, distinguishing ampullary from periampullary tumors is of high importance as this provides significance to the prognosis and resectability; ampullary carcinomas have a more desirable prognosis with five-year survival ranging from 34%-45% [1,3].

Of the primary sites of periampullary carcinoma, the rarest is the duodenum, with the pancreas, ampulla, and bile duct being more common [4]. Patients commonly present in the 6th to 8th decades of life, with equal incidence between men and women. Presenting symptoms are most often right upper quadrant pain, iron-deficiency anemia, weight loss, nausea, vomiting, and infrequently (20%) obstructive jaundice. 

Panendoscopy with histopathological sampling is the diagnostic method of choice. Additional imaging studies such as CT and MRI should be performed to determine the stage of the disease [5]. Imaging may demonstrate various degrees of irregular thickening of the wall or presence of tumor mass in the lumen of the organ, commonly accompanied by obstruction. Early tumors may mimic polyp-like lesions, which can be observed in cases of Gardner’s syndrome since the tumor often originates from an adenomatous polyp [6]. The presence of enlarged lymph nodes, dilated pancreatic and biliary ducts with jaundice, or metastatic lesions may indicate advanced carcinoma [1,4,6].

Whipple’s operation (pancreaticoduodenectomy - PD) with the removal of the adenocarcinoma, pancreatic head, and lymph nodes is the proposed ideal therapy, providing radical oncological management and creates an opportunity for complete recovery [4,6,7]. Resection offers the only possible solution for long-term survival, and without removal of the tumor, the disease is fatal. Local excision should only be considered for patients with small tumors who are surgically unfit or who refuse radical resection [7]. Due to the rare location of the primary tumor, there is a lack of five-year survival results after radical procedures. Results range between 20% and 90% with a negligible number of reports on the efficacy of postoperative chemotherapy [6]. However, it has been suggested that patients with periampullary carcinoma of primary duodenal etiology who have had pancreatoduodenectomy have the longest survival at five years (53%) versus other etiologies (22%) [4]. Factors including resection to negative margins, no lymph node metastasis, and well or moderate tumor differentiation have been shown to have a positive impact on five-year survival [3]. Mortality rate after PD is currently 0%-5% in experienced (>25 procedures/year) centers but is still associated with postoperative complications - the most common of these being postoperative infections and sepsis. Current evidence suggests that postoperative adjuvant chemotherapy and/or radiation does not improve survival and therefore, it is not recommended. 

## Conclusions

Periampullary adenocarcinomas are rare malignancies with an aggressive course of the disease. Our patient presented with atypical chest pain that is not usual with common presentations of these tumors. While cardiac syndromes need to be ruled out, consideration should be given for gastrointestinal etiology of the atypical chest pain, especially in scenarios of unexplained jaundice. As these sites are easily accessible for investigation, early recognition of the disease is paramount. Failure to recognize the disease early may lead to poor prognosis and decreased survival for the patient.
